# N-acetylcysteine suppresses retinal defects in Drosophila models of *SNRNP200*-associated retinitis pigmentosa

**DOI:** 10.1093/hmg/ddag077

**Published:** 2026-08-13

**Authors:** Sara K Mayer, Quinton H Christensen, Hailey McCoy-Munger, Nathaniel P Mohar, Valentina Botero, Seth M Tomchik, Yikang Rong, Arlene V Drack, Lori L Wallrath

**Affiliations:** Interdisciplinary Graduate Program in Genetics, The Graduate College, University of Iowa, 225 S. Grand Ave., Iowa City, IA 52242, United States; Department of Biochemistry and Molecular Biology, Carver College of Medicine, University of Iowa, 51 Newton Rd., Iowa City, IA 52242, United States; Department of Ophthalmology and Visual Sciences, Carver College of Medicine, University of Iowa, 200 Hawkins Dr., Iowa City IA 52242, United States; Department of Biochemistry and Molecular Biology, Carver College of Medicine, University of Iowa, 51 Newton Rd., Iowa City, IA 52242, United States; Department of Ophthalmology and Visual Sciences, Carver College of Medicine, University of Iowa, 200 Hawkins Dr., Iowa City IA 52242, United States; Department of Biochemistry and Molecular Biology, Carver College of Medicine, University of Iowa, 51 Newton Rd., Iowa City, IA 52242, United States; Interdisciplinary Graduate Program in Genetics, The Graduate College, University of Iowa, 225 S. Grand Ave., Iowa City, IA 52242, United States; Department of Biochemistry and Molecular Biology, Carver College of Medicine, University of Iowa, 51 Newton Rd., Iowa City, IA 52242, United States; Department of Neuroscience and Pharmacology, Carver College of Medicine, University of Iowa, 51 Newton Rd., Iowa City, IA 52242, United States; Department of Neuroscience and Pharmacology, Carver College of Medicine, University of Iowa, 51 Newton Rd., Iowa City, IA 52242, United States; Department of Biochemistry and Molecular Biology, Carver College of Medicine, University of Iowa, 51 Newton Rd., Iowa City, IA 52242, United States; Interdisciplinary Graduate Program in Genetics, The Graduate College, University of Iowa, 225 S. Grand Ave., Iowa City, IA 52242, United States; Department of Ophthalmology and Visual Sciences, Carver College of Medicine, University of Iowa, 200 Hawkins Dr., Iowa City IA 52242, United States; Interdisciplinary Graduate Program in Genetics, The Graduate College, University of Iowa, 225 S. Grand Ave., Iowa City, IA 52242, United States; Department of Biochemistry and Molecular Biology, Carver College of Medicine, University of Iowa, 51 Newton Rd., Iowa City, IA 52242, United States

**Keywords:** Drosophila disease model, genetic eye disease, RNA splicing, oxidative stress

## Abstract

Retinitis Pigmentosa (RP) is an inherited retinal degenerative disease that affects 1 in 4000 individuals worldwide and can lead to complete blindness. Early stages of RP involve death of rod photoreceptors via apoptosis, causing loss of peripheral and night vision, which is followed by death of cone photoreceptors, leading to loss of central and daytime vision. Mutations in over 300 genes cause RP. Many of these genes encode retina-specific proteins; however, some encode globally expressed proteins, such as pre-mRNA splicing factors. This study is focused on mutations in the *SNRNP200* gene encoding a core pre-mRNA splicing factor. The pathological mechanisms of *SNRNP200*-associated RP are not well understood and treatments are limited. An approach to study pathogenic mechanisms is to utilize model organisms. Therefore, we developed Drosophila models in which RP-causing mutations were introduced into the *Drosophila melanogaster* orthologue *Snrnp200*. In addition, we used RNAi to knock-down *Snrnp200* in the developing eye. Depletion of *Snrnp200* caused an adult rough eye phenotype due to apoptosis of cells in the retina. When human RP-causing mutations were modeled in Drosophila *Snrnp200*, they resulted in abnormal retinal electrophysiology and defective patterning of photoreceptors. Further analysis of the photoreceptors revealed mitochondrial defects and altered expression of genes related to redox homeostasis. Consistent with these changes, treatment with the antioxidant N-acetylcysteine (NAC) partially suppressed the photoreceptor defects. Taken together, these findings established a new genetic model for studies of splicing-factor associated RP that recapitulates aspects of the human disease and suggests that antioxidants might serve as a treatment for individuals with *SNRNP200*-associated RP.

## Introduction

Retinitis Pigmentosa (RP) is an inherited genetic vision disorder that affects approximately 1 in 4,000 individuals worldwide, with this statistic varying among distinct populations and geographic regions [1–5]. RP is caused by mutations in over 300 genes and can be inherited in an autosomal dominant, autosomal recessive, or X-linked manner [6, 7]. Autosomal dominant inheritance represents 30% of all cases of RP [8]. RP can be syndromic or non-syndromic, depending on the gene and the mutation [9, 10]. Individuals with RP typically present with a loss of peripheral and night vision due to the death of rod photoreceptors via apoptosis [11–13]. This is followed by loss of central and daytime vision due to death of cone photoreceptors [10, 11, 14, 15]. Currently, there are limited treatments available for individuals with RP.

Little is understood about the progressive death of photoreceptors in RP. Different mechanisms of death might occur depending on the gene and the specific mutation [8, 15]. Upon death of the rod photoreceptors, oxygen utilization in the retina is thought to decrease. Thus, one hypothesis is that the death of the rods leads to inappropriate high levels of oxygen supplied by the blood vessel-enriched choroid layer underlying the retinal pigment epithelial cells. The outcome is an abundance of damaging superoxide radicals that modify proteins, nucleic acids, and lipids [11, 16–19]. The retina is among the most sensitive tissues in the body to oxidative stress, owing to its high metabolic,rate and abundance of lipid membranes [20, 21]. ROS can damage lipids through lipid peroxidation, which will affect membranes, resulting in changes in cellular structure and alterations in signal transduction pathways [22]. Studies on rodent retinal degeneration in models and in humans support increased oxidative stress and lipid peroxidation in RP [16, 23–25]. Another hypothesis for the death of cones involves inflammation in which the death of the dying rods trigger an inflammatory response, causing microglia activation and phagocytosis of cones [23, 26]. Other hypotheses posit that death of the rod photoreceptors releases toxic factors that harm the cones or that loss of rods deprives cones of valuable nutrients [26–29]. While these non-mutually exclusive hypotheses have significant support, causal mechanisms of photoreceptor death remain unknown.

Among autosomal dominant RP, 15 to 20% of cases are due to mutations in seven genes that encode pre-mRNA splicing factors (*PRPF3, PRPF4, PRPF6, PRPF8, PRPF31, SNRNP200*, and *RP9*) [8, 9, 30, 31]**.** These factors play a role in pre-mRNA splicing, a process whereby introns are removed from primary mRNA transcripts and exons are united to form processed transcripts [32–34]. The joining of exons occurs through the orchestration of events involving five small nuclear ribonucleoproteins (snRNPs) [35–37]. These snRNPs, U1, U2 and a tri-snRNP complex known as U4/U6.U5 [38], form the core spliceosome—a macromolecular complex of proteins and RNAs that carry out pre-mRNA splicing. Each snRNP is a large complex ranging between 10 000–35 000 kD in size and consisting of small RNAs and several protein subunits [35]. The process of pre-mRNA splicing occurs via two *trans*-esterification reactions [39–41]. While energy, in the form of ATP, is not required for the splicing reaction per se, it is needed for conformational changes such as helicase unwinding of small RNAs during the splicing process [42]. The snRNPs U1 and U2 bind the 5′ splice site and intron branch site of pre-mRNAs, respectively, forming complex A [36, 43–45]. Subsequently, the tri-snRNP U4/U6.U5 associates with the pre-mRNA, forming complex B [46] (Fig. 1A). Several subunits of the snRNP function together to unwind U4 and U6 while also facilitating the replacement of U1 with U6 [38, 47]. Following, U4 exits the spliceosome and U6 associates with U2, forming the activated B complex, also known as the catalytically activated spliceosome [45]. The activated B complex is under tight regulation so that the timing of events in splicing occur properly [48–51].

**Figure 1:**
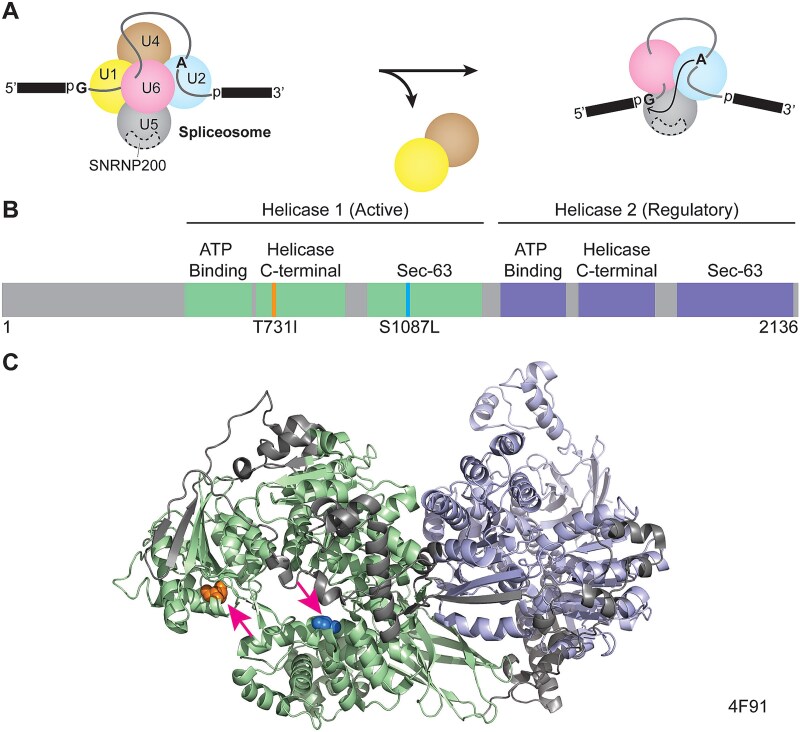
SNRNP200 plays a key role in pre-mRNA splicing. A. SNRNP200 is a subunit of the U5 small nuclear ribonucleoprotein (snRNP). The diagram shows pre-mRNA splicing in which the helicase activity of SNRNP200 unwinds the U4 snRNA from U6 snRNA, allowing U1 and U4 to dissociate from the complex. B. A diagram of the conserved domains of SNRNP200 is shown. T73I and S1087L are the amino acid substitutions caused by mutations in *SNRNP200* studied here. C. A ribbon diagram of the crystal structure of SNRNP200 (PDB: 4F91) is shown with the T731 and S1087 amino acids indicated by in spheres (arrows). The color coding of the protein domains in panel B is maintained in panel C.

Here, we focus on mutations that affect the gene *Small nuclear ribonucleoprotein 200 kd (SNRNP200)*, originally studied in yeast as *Bad response to refrigeration 2* (*Brr2*), that when mutated causes RP (OMIM #610359, RP33) [46, 52, 53]. *SNRNP200* encodes a core component of the U5 snRNP (Fig. 1A) [47, 54]. SNRNP200 possesses RNA helicase activity and is responsible for unwinding U4 and U6 snRNAs, a catalytic step of pre-mRNA splicing [34, 37, 55]. Tight regulation of this helicase activity is vital for timely and proper activation of the spliceosome [48, 49, 56]. SNRNP200 is a DEXH-box ATPase protein family member that possesses two tandem helicase domains [52, 53] (Fig. 1B and C). The first helicase domain possesses active helicase activity and performs RNA unwinding during splicing [57]. The second helicase domain is reported to lack helicase activity; however, it plays a role in the regulation of U5 activity [58, 59] (Fig. 1B and C).

To understand the function of *SNRNP200* in the retina and identify potential treatments for *SNRNP200-*associated RP, we developed *Drosophila melanogaster* models. Drosophila has been used to study human RP, including autosomal dominant RP caused by mutations in the pre-mRNA splicing factor genes *PRPF8* and *PRPF31* [60–62]. Drosophila models allow for developmental and genetic analyses of retinal dysfunction. Despite the differences in eye morphology, many of the developmental processes and physiology are shared between humans and fruit flies [63, 64]. *D. melanogaster* has a single orthologue of human *SNRNP200* that encodes a protein with a remarkable 74% amino acid identity and 86% similarity at the amino acid level. This orthologue was identified by an ethyl methanesulfonate mutagenesis screen for lethal genes on the third chromosome [65]. Six non-complementing mutations mapped to the salivary gland polytene chromosome subdivision 72A, and the gene was originally named *lethal (3)72Ab* [65]. Recently, the gene was labeled *Brr2* in Flybase after the yeast orthologue. However, for simplicity and logic, we will refer to the fruit fly orthologue as Drosophila *Snrnp200*.

Here, we determine the effects of depletion of *Snrnp200* in the developing Drosophila retina. In addition, we use CRISPR mutagenesis to generate point mutations that cause dominant RP in humans. We find that depletion of *Snrnp200* in the retina causes increased cell death and a rough eye phenotype [66]. We show that dominant disease-causing mutations introduced into Drosophila *Snrnp200* lead to abnormal patterning of photoreceptors and loss of photoreceptor function. At the cellular level, these defects correlate with abnormal mitochondria in photoreceptors and altered redox gene regulation, suggesting a lack of cellular energy homeostasis. These abnormal retinal phenotypes are partially suppressed by treatment with the antioxidant N-acetylcysteine (NAC), suggesting the possibility that altered redox plays a role in the pathogenesis of *SNRNP200*-associated RP and that antioxidants might serve as treatments.

## Results

### A rare mutation in *SNRNP200* causes RP

We report on a mutation in *SNRNP200* that was identified in a child who presented at age six with night blindness. At age nine, visual acuity was 20/20 in both eyes; however, fundus photographs showed a waxy pallor of the optic discs and attenuated retinal arterioles in both eyes, which are characteristics of RP (Fig. 2A) [15]. Full-field ERG recordings that measure the electrical activity of the retina revealed an absence of rod photoreceptor responses with attenuated, yet recordable cone responses in both eyes (Fig. 2B), consistent with the presentation of the self-reported night blindness. Molecular genetic testing utilizing a retinal disease gene panel on an exome sequencing platform (Precision Genetics, Greenville, SC) revealed heterozygosity of a single nucleotide change in the *SNRNP200* gene that is predicted to be pathologic (c.2192C > T; pT731I). No other potential disease-causing gene candidates were identified. This base change was verified by Sanger sequencing performed at the University of Iowa Shivanand R. Patil Cytogenetics and Molecular Laboratory (Supplementary Fig. 1). The amino acid altered (T731) maps to the active helicase domain of the SNRNP200 protein (Fig. 1B-C, orange). To the best of our knowledge, at the time, the *SNRNP200* variant was not reported in either public genomic sequence databases or the literature. However, a recent in-depth clinical phenotyping and genetic characterization of individuals with *SNRNP200*-associated RP identified a second individual with this mutation [67]. In our case study, neither parent carries the variant, strongly suggesting a *de novo* mutation. Given that this *SNRNP200* mutant allele is rarely found in hundreds of thousands of genome sequences (Clinvar, gnomAD, access date 10/2025), it was designated ‘*Bigfoot* (*Bf*)’, as it is rarely seen.

**Figure 2:**
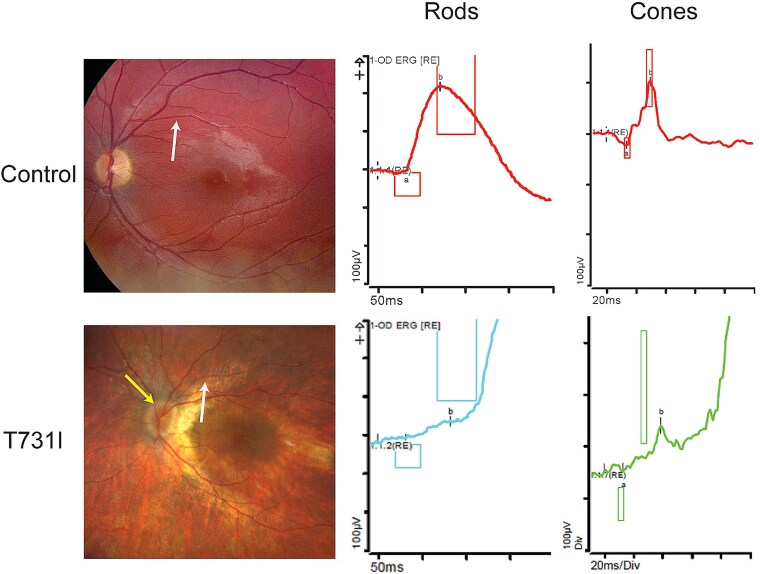
The retinal phenotypes of an individual with a mutation in the *SNRNP200* gene are shown. A. Fundus photos of a retina from an age-matched control individual and an individual with a mutation in *SNRNP200* that produces the T731I amino acid substitution (Fig. 1) are shown. The white arrows indicate arterioles, which are thinner in the retina of the individual with RP compared to that of the control. The yellow arrow points to a waxy pallor of the optic disc which is a characteristic of RP. B. Electroretinogram (ERG) waveforms of a control individual and an individual with a mutation in *SNRNP200* that causes the T731I amino acid substitution are shown on the right. To measure the function of the rod photoreceptors, ERGs were performed following dark adaptation. To measure the function of cone photoreceptors, ERGs were performed following light adaptation. Boxes outline the range observed for unaffected control individuals in both plots.

Mutations in the *SNRNP200* gene encoding a component of the spliceosome are known causes of RP [31]. Approximately 30% of the cases of RP are caused by dominant mutations, such as the one reported here [8, 9]. There are currently 17 known pathogenic mutations in *SNRNP200*, of which, 15 are missense mutations and two are predicted loss–of–function mutations. Fifteen of these mutations cluster within the active helicase domain, while one is in the regulatory domain, and one in the 5′ untranslated region (Clinvar, gnomAD, access date 6/2026). *SNRNP200* is expressed in nearly all tissues, yet mutations only cause retinal-specific defects by mechanisms that are not well understood.

### Depletion of *Snrnp200* causes a rough eye phenotype in Drosophila

To understand the role of *SNRNP200* in the retina, we employed Drosophila due to the high level of conservation between the human and fruit fly protein, and the genetic tools available for retinal-specific expression in flies [68–70]. Given that deletion of the *Snrnp200* gene is homozygous lethal in Drosophila, as well as other organisms [65], we assess the consequences of *Snrnp200* RNAi knock-down specifically in the developing Drosophila eye. To accomplish this, a *Snrnp200* RNAi transgene was expressed under the control of an upstream activating sequence (UAS) that binds to the yeast Gal4p transcription factor [69]. Adults from a *Snrnp200* RNAi stock (Bloomington Stock Center, #34024) were crossed to adults from a stock expressing the yeast transcription factor Gal4p in cells posterior to the morphogenic furrow in the developing retina (GMR-Gal4, Bloomington Stock Center, #1104). The resulting progeny possessed a rough eye phenotype (Fig. 3A, top). This is a well-characterized phenotype that is an indicator of retinal cell death [66]. The rough eye phenotype was observed in 70% of the males and 100% of the females (Fig. 3B, left). By contrast, no adults expressing control RNAi for an unrelated gene had a rough eye (Fig. 3A, top row), indicating specificity of the phenotype for *Snrnp200* RNAi and not activation of the RNAi pathway (Bloomington Stock Center, # 33681). These data suggest that *Snrnp200* is required for eye development. We also tested if the rough eye phenotype was dependent on light. This was done because some models of retinal degeneration in Drosophila are light-induced [60, 71, 72]. Adults with the *Snrnp200* RNAi were crossed to adults of the GAL4 expressing stock, and the progeny were cultured completely in the dark. Despite the lack of light, the adult progeny had similar levels of rough eye as those raised in the light (Supplementary Fig. 2), indicating dependence of the phenotype on the genetic perturbation as opposed to culture conditions.

**Figure 3:**
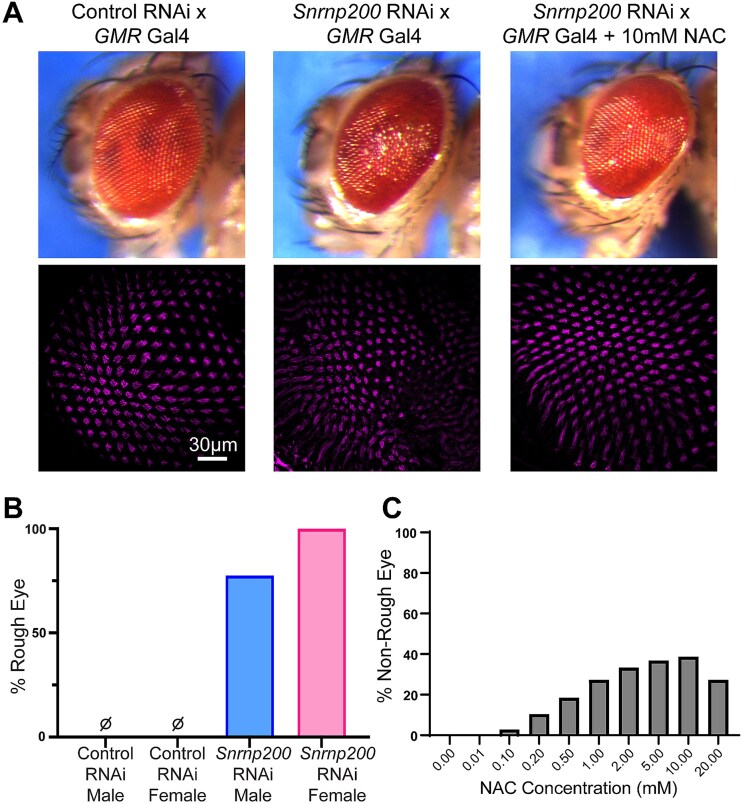
Knock-down of *Snrnp200* during Drosophila eye development results in a rougheye adult phenotype that is suppressed by N-acetylcysteine (NAC). A. Eye-specific RNAi knock-down of *Snrnp200* was achieved using the GMR-GAL4 driver stock. Adults of the GMR-GAL4 driver stock were crossed to adults expressing either a control RNAi or a *Snrnp200* RNAi and cultured on food with either 10 mM NAC or vehicle alone. The eyes of the adult progeny are shown in the top row. Retinas dissected from the adult progeny were stained with phalloidin (marker of F-actin) and are shown in the second row. B. Graph of the percent rough eye in male and female adults expressing either a control RNAi or a *Snrnp200* RNAi is shown. The number of adults scored ranged from 16 to 136 per genotype. C. Adults with GMR-GAL4 were crossed with adults possessing an RNAi against *Snrnp200* and cultured in food containing various amounts of NAC as indicated. The graph shows the percentage of non-rough eye adult progeny for each concentration of NAC, demonstrating dosage dependance, with the most effective concentration of NAC at 10 mM. The number of adults scored was 20–120 per concentration.

To examine how *Snrnp200* depletion altered cells within the retina, phalloidin staining was performed. The compound adult Drosophila eye is composed of ~ 800 repeating units called ommatidia [73]. Within each ommatidium is a cluster of eight photoreceptors arranged in a stereotypical pattern that can be easily visualized by phalloidin staining. The staining revealed that the rough eye phenotype was due to abnormal organization of the ommatidia (Fig. 3A, bottom row). Flies expressing RNAi against *Snrnp200* had abnormal ommatidia patterning, unlike control flies which had ommatidia arranged in an organized pattern. Disruptions in ommatidia pattern formation have been observed in mutants in genes affecting cell–cell signaling, suggesting cell-to-cell contact might be altered by reduced *Snrnp200* [71, 74].

### Depletion of *Snrnp200* in the Drosophila eye causes a rough eye phenotype that is suppressed by N-acetylcysteine (NAC) treatment

RP has been associated with oxidative stress [75–80]. To determine if redox regulated genes were activated by knock-down of *Snrnp200*, we performed qRT-PCR to determine the mRNA levels of candidate genes. Genes activated by oxidative stress have been well-studied in Drosophila [81]. RNA was extracted from heads of seven-day-old adults expressing either the control RNAi or the *Snrnp200* RNAi. The results showed that the mitochondrial enzyme gene *Glutamate oxaloacetate transaminase 2* (*Got2, CG10383)* encoding a hydrolase, *4-hydroxyphenylpyruvate dioxygenase* (*Hpd*)*, phosphoserine transaminase* (*Psat*), *serine hydroxymethyl transferase* (*Shmt*)*,* and *CG12224* were significantly upregulated in the *Snrnp200* knock-down flies relative to the control, consistent with oxidative stress (Fig. 4, top two rows). However, *Multidrug resistance protein 4* (*Mrp4*), *Glutathione S transferase Z2* (*GstZ2*), and *L-threonine 3-dehydrogenase* (*Tdh*) were not up-regulated (Fig. 4, bottom row). Thus, some key genes known for a role in detoxification of an oxidative environment were up-regulated.

**Figure 4:**
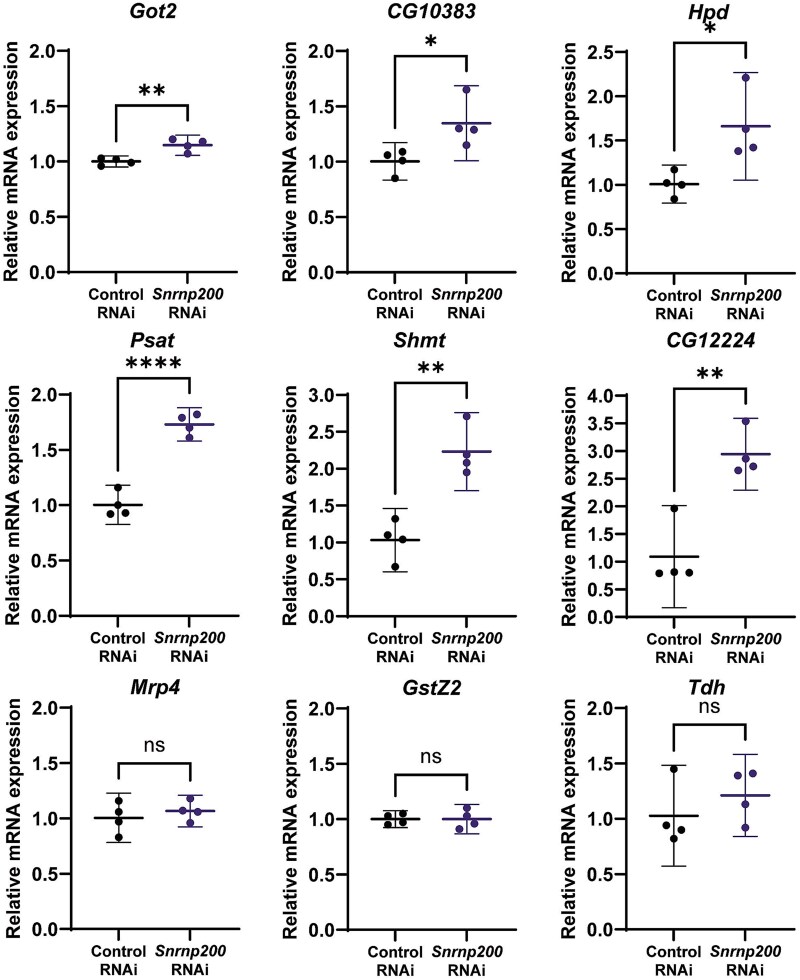
Knock-down of *Snrnp200* causes up-regulation of genes known to be induced by compounds that cause oxidative stress. qRT-PCR was performed on RNA isolated from four sets of ~ 25 adult heads per genotype. The ΔΔCt method was used to generate fold changes in relative expression level of each gene following normalization to reference genes *GAPDH* and *ribosomal protein L32*. Error bars represent standard deviation. Expression fold changes were compared using a Student’s T-test for each gene. ^*^, *p* < 0.05; ^**^, *p* < 0.01; ^****^, *p* < 0.0001.

Given the up-regulation of redox related genes, we wondered if antioxidant treatment would suppress the rough eye phenotype caused by depletion of *Snrnp200*. NAC provides cysteine, a thiol-containing amino acid with a thiol side chain that is a precursor in the synthesis of the glutathione (GSH) [82]. Adult flies possessing either the RNAi transgene against either *Snrnp200* or a control transgene were mated to adult flies possessing the eye-specific GAL4 transgene in vials containing food supplemented with either NAC or vehicle (water) alone. The progeny consumed the food with or without NAC throughout development. The resulting adult progeny were collected upon emergency from their pupal cases and aged seven days in vials lacking NAC and then their eyes were examined visually under the dissecting scope. 100% of the females exhibited a rough eye phenotype; therefore, females were used for scoring suppression by NAC. Suppression of the rough eye phenotype showed a linear dosage response to NAC treatment up to a concentration of 10 mM, at which ~ 40% of the females showed suppression (Fig. 3). Concentrations greater than 10 mM appeared to decrease adult survival.

### The rough eye phenotype is caused by apoptosis and reduced by NAC treatment

To determine if the rough eye phenotype was caused by increased cell death in the developing eye, eye/antennal imaginal discs of third instar larvae were assayed for apoptosis. Imaginal discs are clusters of epidermal cells present in the third instar larvae which differentiate into organs in the adult fly [73, 83]. The eye/antennal imaginal disc is a gourd-shaped structure with the antennal disc at the anterior end and the eye disc at the posterior end [84]. The morphogenetic furrow transitions from the posterior towards the anterior of the eye disc as the larva progresses through the third instar larval stage. As the morphogenic furrow migrates, the cells behind the furrow differentiate into a retinal cell fate [83, 85]. Cells that will differentiate into photoreceptors can be detected with antibodies to embryonic lethal abnormal vision (elav), an RNA-binding protein that plays a role in photoreceptor differentiation [86–88]. Apoptosis in the larval eye/antennal disc can be detected by staining with antibodies that recognize cleaved Death Caspase 1 (c-DCP1) [89]. When using the GMR-GAL4 driver, only cells posterior to the morphogenetic furrow express the GAL4 protein. We found that eye/antennal imaginal discs depleted for *Snrnp200* exhibited increased c-DCP1 signal intensity posterior to the morphogenic furrow compared to controls (Fig. 5A and B). Thus, depletion of *Snrnp200* from cells in the retina increases cell death, consistent with an essential role in many different cell types and organisms [90–92].

**Figure 5:**
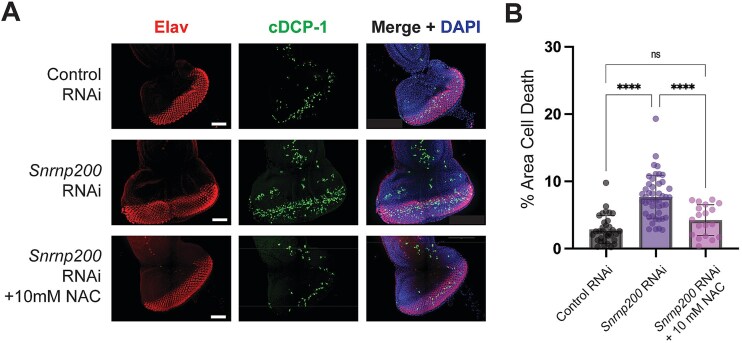
Depletion of *Snrnp200* during eye development increases apoptosis that is suppressed by NAC. A. Adults expressing GMR-GAL4 were crossed to adults possessing either a control RNAi or *Snrnp200* RNAi on food with 10 mM NAC or vehicle only. The eye/antennal discs from the larval progeny were immunostained with the neuronal marker embryonic lethal abnormal vision (elav) (red), the apoptotic marker cleaved death caspase 1 (cDCP-1) (green), and DAPI (blue). The GMR-GAL4 driver expresses Gal4 in differentiated photoreceptors marked with elav. B. The intensity of the cDCP-1 immunofluorescence within the region posterior to the morphogenic furrow was quantified and graphed. The number of eye discs quantified ranged from 19 to 46 per genotype. Error bars represent the standard deviation. A Šídák’s multiple comparisons test was used to determine statistical significance (GraphPad prism software, GraphPad, LLC Boston, MA USA). Ns, non-significant; ^****^, *p* < 0.0001.

Consistent with our previous findings of NAC suppressing the rough eye phenotype, larvae raised on NAC-containing food showed a reduction in c-DCP1 staining relative to controls (Fig. 5A and B). Thus, NAC treatment reduced cell loss via apoptosis, further suggesting NAC treatment might benefit individuals with *SNRNP200*-associated RP.

### *Snrnp200* mutant alleles have retinal-specific defects

To determine the consequences of disease-causing point mutations in *SNRNP200*, we used CRISPR mutagenesis [93]. We selected two RP-causing mutations that alter conserved single amino acids within the active helicase domain in SNRNP200 and modeled them in Drosophila (Fig. 1B and C). We generated a point mutation that causes the T731I substitution identified in the patient under study here. In addition, we generated a mutation that causes the S1087L amino acid substitution, one of the most common pathogenic mutations in *SNRNP200* (ClinVar, National Institute of Health, accession number VCV000007928.47, access date 6/2026), with an allele frequency of 5.58 × 10^−6^ (gnomAD, Broad Institute, https://gnomad.broadinstitute.org/, access date 6/2026). Given the high degree of amino acid sequence conservation between the human and Drosophila SNRNP200 protein, the amino acids altered in disease are conserved in fly Snrnp200 and remarkably, the amino acid numbering is identical. The mutations introduced into Drosophila *Snrnp200* were confirmed using Sanger sequencing and no other base changes within the coding region and 300 bp flanking the gene were identified.

We performed genetic complementation analyses with these two novel *Snrnp200* alleles with the originally isolated *Brr2* (*Snrnp200*) null alleles [94]. As shown in Supplementary Table 1, flies homozygous for the *Brr2* null alleles or *trans*-heterozygous for the null alleles die early in the development, either at the embryonic or first instar larval (L1) stage. This is consistent with prior reports [65, 94]. The *Snrnp200^S1087L^* allele produces viable and fertile adults with no visible defects either as homozygotes or *trans*-heterozygous with either of the *Brr2* null alleles. By contrast, the *Snrnp200^Bf^* allele causes embryonic lethality either as homozygotes or when *trans*-heterozygous with either of the *Brr2* null alleles. Interestingly, when the *Snrnp200^Bf^* allele was made *trans*-heterozygous with the *Snrnp200^S1087L^* allele, the resulting progeny showed delayed development, ultimately surviving to the third instar larval and pupal stages. Surprisingly, eight percent of these *Snrnp200^S1087L/Bf^* mutants survive to adulthood. These genetic complementation results indicate that the homozygous lethality of the *Snrnp200^Bf^* allele is unlikely to be due to a second site mutation generated during the CRISPR mutagenesis. Our findings also suggest that the *Snrnp200^S1087^* allele is a weak loss-of-function allele and that *Snrnp200^Bf^* is a dominant allele, as the combination of both alleles causes lethality at early stages.

### *Snrnp200* mutants have phototransduction defects

The *SNRNP200* point mutations studied here cause non-syndromic, autosomal dominant RP. Therefore, we wondered if the Drosophila models, which outwardly do not appear to have any abnormalities, possessed retinal defects. To test for this, we performed ERG analysis on adult flies. ERGs provide a powerful tool that has been used for over five decades to analyze phototransduction in living flies [95–98]. Analyses of a plethora of visual system mutants have revealed key findings related to G-protein coupled receptors and intercellular signaling, which have established paradigms for other systems and organisms [99]. ERGs provide a quantitative measurement of collective photoreceptor activity. For ERG recordings, an electrode records the compound field potential from photoreceptors and downstream neurons in response to flashes of light. A waveform with reproducible characteristics is captured in real-time [95, 97, 98]. Transient spikes occur at the onset and offset of a light flash, called on-transients and off-transients, respectively (Fig. 6). These correspond to the hyperpolarization potentials of postsynaptic laminar neurons. A sustained potential during the light stimulus results from the depolarization of photoreceptors.

**Figure 6:**
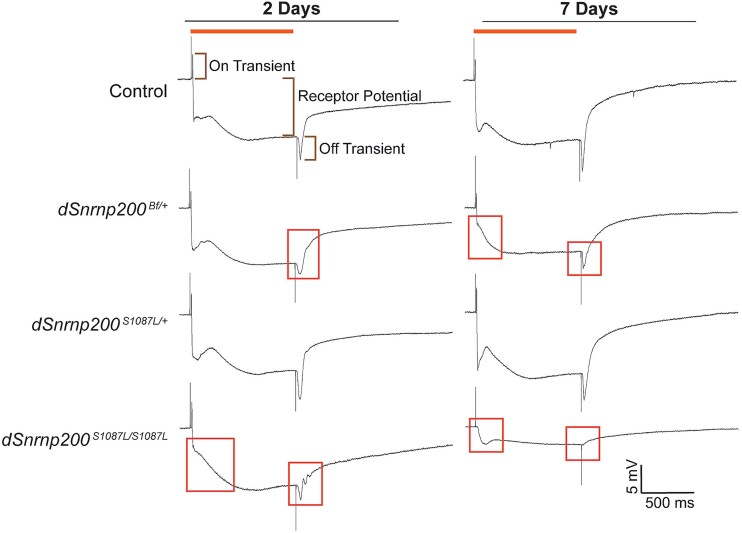
ERG waveforms of drosophila models of *SNRNP200*-associated RP show progressive loss of photoreceptor function. The duration of the orange light pulses are indicated by the orange bars. Representative ERG waveforms of age-matched control adults (*w^1118^*) are shown at the top. Brackets indicate mass receptor potential that arises from the photoreceptors. The on and off transients are generated by voltage from the synaptic activity to the lamina (brackets). Red rectangles indicate regions of waveform abnormalities. ERG waveforms produced from the mutant *Snrnp200* alleles are shown below. Three to six adults were assayed per genotype. No statistical difference in receptor potential was found between the genotypes at two or seven days of age; however, there were abnormalities in the shape of the waveforms indicative of malfunction (boxes).

First, ERGs were performed to measure the collective photoreceptor potential in response to a flash of orange light (Fig. 6) [97]. The ERG waveforms generated from the control (*w^1118^*) flies were compared to those of the *Snrnp200* mutants of the same age and raised simultaneously under similar conditions. All flies possessed a mutation in the *white* gene, which encodes a pigment transporter [100–102], such that light interference by naturally occurring eye pigments was avoided. When stimulated by orange light, the control flies showed the anticipated waveform (Fig. 6). By contrast, the *Snrnp200^Bf^* heterozygotes showed abnormal waveforms, with slow off-transients even in two-day-old adults (Fig. 6, red boxes). The waveforms of *Snrnp200^S1087L^* heterozygotes appeared normal; however, the homozygotes showed abnormal off-transients (Fig. 6, red boxes). Slow off-transients typically indicates abnormal input of the signal to the underlying lamina.

Second, we performed ERGs to measure the Prolonged Depolarizing Afterpotential (PDA), a state in which the maximal receptor depolarization and desensitization are generated by multiple flashes of blue light (460–490 nm). The photoreceptors remain in a depolarized state long after blue light is no longer present [103]. During PDA, Rhodopsin 1 (Rh1), which is the major photopigment specifically present in photoreceptors R1–6, is converted to metarhodopsin (Fig. 7A). PDA can be interrupted by flashes of orange to red light (580–640 nm) in which the metarhodopsin is reconverted to Rh1. A lack of PDA is an indication of reduced phototransduction in photoreceptors R1–6.

**Figure 7:**
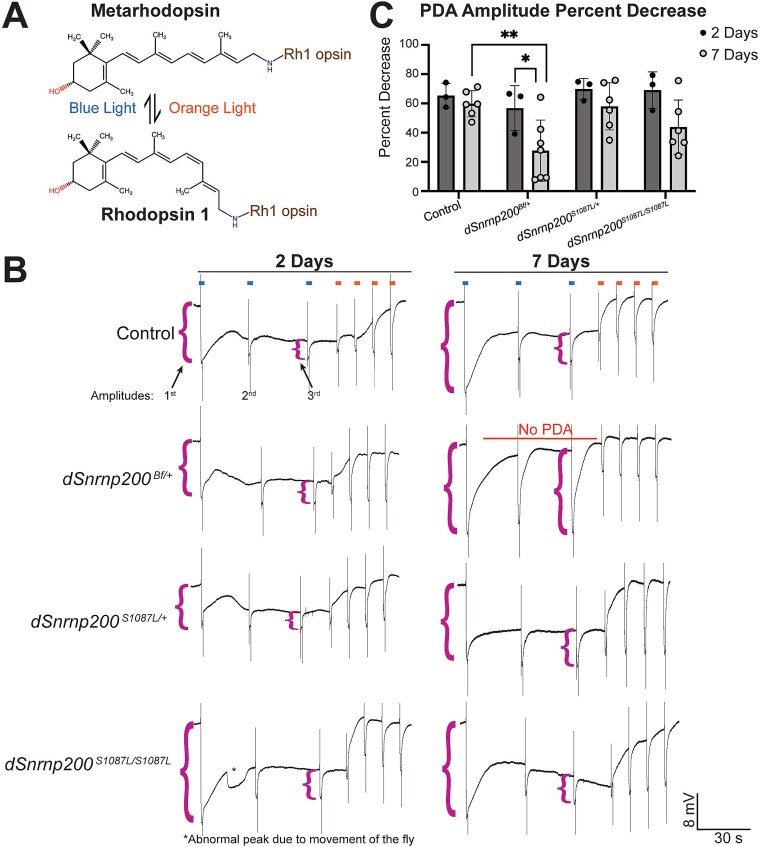
Prolonged depolarizing afterpotential (PDA) analysis shows progressive decline of photoreceptor function in drosophila models of *SNRNP200*-associated RP. A. Diagram of the reversible reaction of rhodopsin 1 to Metarhodopsin by blue light and vice versa with orange light. B. Representative ERG waveforms of the control (*w^1118^*) and *Snrnp200* mutants. Pulses of light are indicated by small blue and orange boxes at the top. The magenta brackets indicate receptor potential responses to the first and third blue light, followed by oranges pulses for recovery. The height of the magenta brackets represents the change in amplitude from baseline to depolarization of the photoreceptors upon light stimulation. The dark orange line above indicates the loss of PDA at seven days of age. C. The graph shows the percent decrease in the photoreceptor potential between the first and third blue light pulses. Higher percentages indicate proper function of photoreceptors R1–6; lower percentages indicate that photoreceptors R1–6 are not functioning properly. Three to six adults were assayed per genotype. Error bars represent standard deviation. Šídák’s multiple comparisons test was used to determine statistical significance (GraphPad prism software, GraphPad, LLC Boston, MA USA). ^*^, *p* < 0.05; ^**^, *p* < 0.01.

PDA analysis provided insights into the molecular dysfunction caused by the *Snrnp200* mutants. Multiple flashes of blue light were delivered to adult eyes, converting Rh1 to metarhodopsin (Fig. 7A). This was followed by multiple flashes of orange light to reconvert the metarhodopsin to Rh1. Measurements of the percent decrease in receptor potential amplitude after PDA were plotted (Fig. 7B and C). Seven-day-old *Snrnp200^Bf^* adults had significantly reduced PDA compared to controls. The PDA results showed a progressive loss of photoreceptor function; seven-day-old *Snrnp200^Bf^* adults had reduced PDA compared to two-day-old adults. Thus, the fly models recapitulated several aspects of the human disease: [1] retinal defects in an otherwise normally developed organism, [2] a progressive reduction in phototransduction with age, and [3] defective function of photoreceptors R1–6, the rod-like photoreceptors.

### *Snrnp200* mutants have wild-type levels and anticipated localization of rhodopsin 1 (Rh1)

The loss of PDA in the *Snrnp200^Bf^* heterozygotes suggested that the rod-like photoreceptors (R1–6) were not functioning properly. This prompted us to examine the levels of Rh1 in the *Snrnp200* mutants. Rh1 is responsible for phototransduction in photoreceptors R1–6 [104]. To examine Rh1 levels, protein extracts were made from the heads of seven-day-old flies. An antibody specific for Rh1 was used for western analysis. REVERT total protein stain (LI-COR, Lincoln, NE) was used for protein normalization among samples. The western analysis showed that the eyes of *Snrnp200* mutants have similar levels of Rh1 as the control (Supplementary Fig. 3A and B). We then considered the possibility that Rh1 was mis-localized in the *Snrnp200* mutants. To determine if this was the case, retinas were dissected and stained with DAPI and antibodies to Rh1. No overt differences in Rh1 localization was observed between the host stock control (*w^1118^*) and the *Snrnp200* mutants (Supplementary Fig. 3C); however, it is possible that subtle differences in localization and/or Rh1 aggregation might be missed by light microscopy. Thus, a loss of Rh1 protein or gross Rh1 mis-localization did not appear to be the cause for the altered PDA.

### *Snrnp200* mutants have photoreceptor morphological defects that are partially improved upon treatment with N-acetylcysteine

Given that Rh1 levels were not altered in the *Snrnp200* mutants, we wanted to assess the arrangement of the photoreceptors in the adult retina as disorganization is known to alter function [105, 106]. Drosophila photoreceptors are organized in cell clusters of eight, with photoreceptors R1–6 in an outside ring and photoreceptors R7 and R8 centrally stacked with R7 on top of R8 (Fig. 8A). To examine the organization of the photoreceptors, we stained whole-mount adult fly retinas with phalloidin, which binds to filamentous actin (F-actin) in the cytoskeleton of the phototransduction microvilli of Drosophila [107]. Flies were aged seven days, and the retinas were dissected, fixed with formaldehyde, and stained with phalloidin*.* Staining of F-actin in the *w^1118^* control fly retinas showed the typical rosette pattern of R1–6 arranged around R7 and R8 [108] (Fig. 8B). However, *Snrnp200^Bf/+^* and *Snrnp200^S1087L^* homozygotes showed abnormal photoreceptor structure and arrangement, forming a ring-like arrangement (Fig. 8B). An analysis of the distance between the centers of neighboring ommatidia revealed increased spacing between photoreceptor clusters in *Snrnp200^Bf/+^* and *Snrnp200^S1087L^* homozygotes compared with the *w^1118^* control (Fig. 8C). When flies were raised on food containing 10 mM NAC, the ring-like arrangement showed partial improvement in *Snrnp200^S1087L^* homozygotes yet persisted in *Snrnp200^Bf/+^* heterozygotes (Fig. 8B-C). Following NAC treatment, the average distance between ommatidia decreased in both *Snrnp200^S1087L^* homozygotes and *Snrnp200^Bf^* heterozygotes compared to the untreated flies (Fig. 8B-C). Thus, NAC treatment alleviated the abnormal spacing of the photoreceptors in the mutants; however, it had minimal effects on the abnormal photoreceptor arrangement.

**Figure 8:**
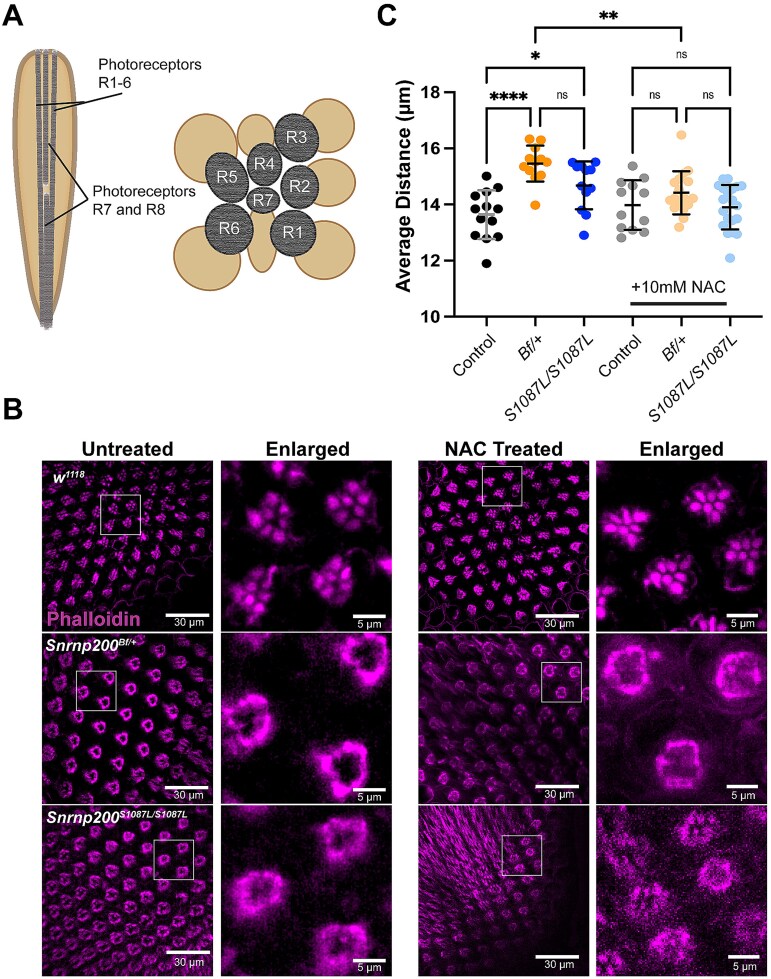
The *Snrnp200* mutations cause abnormal photoreceptor cell morphology that is partially suppressed by NAC. A. The diagram of the organization of the drosophila photoreceptors within each ommatidium. A cross-section showing the stacking of photoreceptors R7 and R8 is on the left. A top-down view of photoreceptors R1–7 is shown on the right. B. Phalloidin staining of F-actin in the photoreceptors is shown. Eyes from untreated adults are shown on the left and NAC treated on the right. The *Snrnp200* photoreceptors show improper patterning and form a ring. Only the *Snrnp200^S1087L/S1087L^* flies show partial suppression of the defect by NAC. C. The graph shows the quantification of the average distance between the center of a photoreceptor cell cluster to the center of an adjacent photoreceptor cell cluster for each genotype. Twelve to 20 measurements from three adults were analyzed per genotype. Error bars represent standard deviation. Šídák’s multiple comparisons test was used to determine statistical significance (GraphPad prism software, GraphPad, LLC Boston, MA USA). Ns, non-significant; ^*^, *p* < 0.05; ^****^, *p*< 0.0001.

To determine if the spacing defects observed in the adult eye of the *Snrnp200* mutants could be caused by cell death in the developing eye, eye/antennal discs of third instar larvae were fixed and stained with DAPI and antibodies to elav and c-DCP1. While the *Snrnp200^S1087L^* homozygotes had abnormally organized photoreceptors, only the *Snrnp200^Bf/+^* heterozygotes had significantly increased staining in the eye disc compared to controls (Supplementary Fig. 4). NAC treatment partially reduced the apoptosis seen in *Srnp200^Bf/+^* heterozygotes (Supplementary Fig. 4). These results indicate that increased cell death is unlikely to account for the unusual ring-like arrangement of the photoreceptors.

To better understand the abnormal photoreceptor arrangement, we performed transmission electron microscopy (TEM) on adult retinas. The adults were aged under similar conditions as those used for the ERGs and phalloidin staining, dissected, fixed, and prepared for EM. Retinas from the *w^1118^* control flies at five days of age showed a typical photoreceptor arrangement with healthy photoreceptors and membrane-enriched rhabdomeres (Fig. 9A). By 11 days old, *w^1118^* flies had limited retinal degeneration, likely due to white-eyed flies having light sensitivity. By contrast, the *Snrnp200* mutants showed thin, misshaped photoreceptors with enlarged interphotoreceptor spaces, consistent with the phalloidin staining observed at the light microscope level (Figs. 8B and 9A). Closer examination of the EMs revealed an increased number and size of mitochondria in the photoreceptors of the mutant *Snrnp200* adults (Fig. 9A-C). Furthermore, the mitochondria in the *Snrnp200* mutants showed abnormal cristae, which is an indicator of low energy production and redox imbalance [109, 110]. Analysis of CO_2_ production of the mutants indicated that the *Snrnp200* mutants had increased respiration, which could be a result of mitochondrial dysfunction (Fig. 9D).

**Figure 9:**
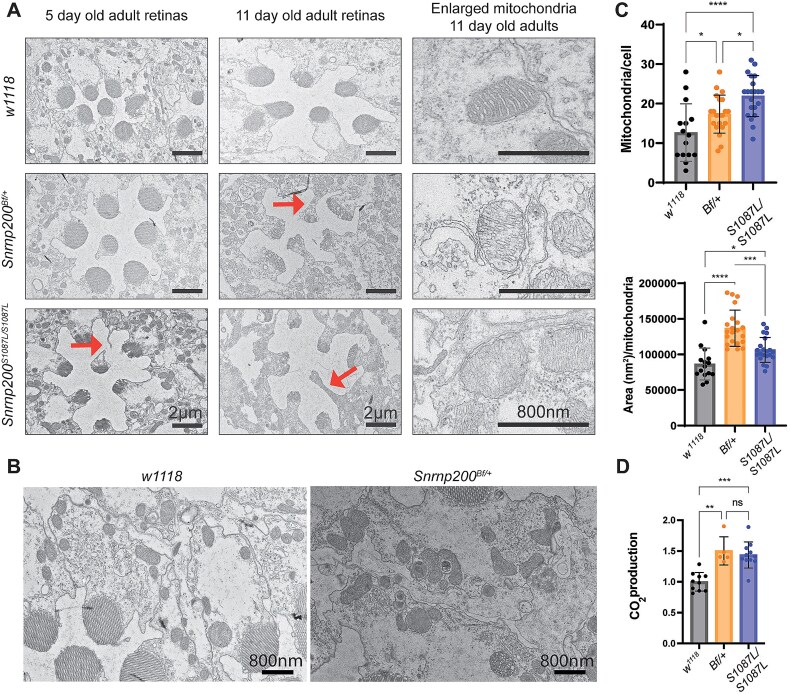
Transmission electron microscopy (TEM) revealed that the *Snrnp200* mutants have abnormally shaped photoreceptors with increased number and size of mitochondria. A. Images of representative photoreceptor clusters from retinas of five- and eleven-day-old adults for each genotype are shown. The arrows denote degenerating the photoreceptor stalks that exhibit deterioration. An enlarged image of a representative mitochondria from the retinas of 11-day-old adults for each genotype is shown in the right column. The *Snrnp200* mutants exhibit progressive photoreceptor degeneration with enlarged spacing and dysmorphic mitochondria. B. Representative images used to quantify the size and number of mitochondria. C. Quantification of number of mitochondria per photoreceptor and the size of mitochondria is shown for each genotype. 15 to 21 mitochondria from three individuals were analyzed per genotype. Error bars represent standard deviation. Šídák’s multiple comparisons test was used to analyze mitochondria data. Ns, non-significant; ^*^, *p* < 0.05; ^***^, *p* < 0.001; ^****^, *p* < 0.0001. D. Carbon dioxide production was measured in *Snrnp200* mutant and control adult flies. *Snrnp200* mutants have increased CO_2_ production compared to control. Normalized CO_2_ production values (n = 5 to10 animals/genotype) were compared between the *Snrnp200^Bf/+^, Snrnp200*^S1087L/S1087L^, *w^1118^* genotypes. Error bars represent standard error. Significance was determined using a one-way ANOVA with Tukey’s multiple comparisons test. Ns, non-significant, ^**^, *p* < .01, ^***^, *p* < .001.

Specific Drosophila genes, when mutated, have been associated with increased oxidative stress and deformation of ommatidia, including *crumbs, Mitochondrial assembly regulatory factor* (*Marf*), and *Optic atrophy 1* (*Opa1*) [71, 111, 112]. Therefore, we perform qRT-PCR to determine if the mRNAs from these genes were altered in the *Snrnp200* mutants using primers specific for these genes (Supplementary Table 2). We discovered that expression of *crumbs* and *Marf* was decreased in *Snrnp200^Bf/+^* adult flies (Supplementary Fig. 5). By contrast, there was no change in expression in *Snrnp200^S1087L/S1087L^* flies, which exhibit a weaker phenotype than the *Snrnp200^Bf/+^* flies. Neither mutant showed a change in expression of *Opa1* (Supplementary Fig. 5). Thus, the severe defects observed in *Snrnp200^Bf/+^* flies might be due to down-regulation of *crumbs and Marf,* as these mutants have similar phenotype defects in the retina.

## Discussion

*SNRNP200* orthologues in eukaryotic species are ubiquitously expressed, consistent with a vital role in pre-mRNA splicing, which is needed in all cell types [31, 90, 113]. As such, *SNRNP200* is essential for viability in many cell types and organisms. CRISPR/Cas9 loss-of-function analyses performed in 1186 cell lines showed that in all cell types tested, *SNRNP200* was required for viability (DepMap, Broad Institute, https://depmap.org/portal/, access date 6/2026). In addition, RNAi knock-down of *SNRNP200* in 695 of 710 cell lines caused lethality (DepMap). Female and male mice homozygous for a *Snrnp200* knock-out allele exhibit a completely penetrant pre-weaning lethal phenotype and a lack of organ development (Mouse Phenotyping Consortium, https://www.mousephenotype.org/data/genes/MGI:2444401, access date 6/2026). Female heterozygotes for the knock-out allele show increased monocyte number; males exhibit an enlarged heart, exophthalmos, and impaired corneal blink reflexes compared to age-matched controls. Therefore, *SNRNP200* function is essential at the cellular and organismal level.

Our studies demonstrate that eye-specific depletion of *Snrnp200* causes apoptosis during eye development, resulting in an adult rough eye phenotype in which photoreceptor clusters are disorganized (Fig. 3). We do not know why rough eye phenotype was more prevalent in females than in males (Fig. 3B). It is worth noting that sex-specific differences have been observed in mouse models of RP [25, 114]. A faster progression of retinal degeneration in multiple mouse models was observed upon sexual maturation and attributed to female hormones [114]. Ovarian removal reduced the retinal degeneration to that of males, and estradiol treatment enhanced deterioration in females. Interestingly, Drosophila eye development is regulated by the major steroid hormone 20-hydroxyecdysone [115], and ia genetic interaction between *Snrnp200* mutations and ecdysone signaling is possible.

In this study, suppression of the rough eye phenotype we observed with NAC treatment, suggesting oxidative stress might play a role. Despite our best efforts, we did not find evidence of increased oxidative stress in third instar eye imaginal discs from flies expressing *Snrnp200* RNAi compared to controls. Thus, it is unclear if the antioxidant functions of NAC are responsible for the suppression. NAC has non-antioxidant functions, including that of an anti-inflammatory by down-regulating the NFkappaB pathway [82, 116].

In contrast to knock-down of *Snrnp200*, the dominant RP-causing point mutations modeled in *Snrnp200* do not cause an adult rough eye phenotype. Instead, these mutations cause a progressive degeneration of photoreceptors (Figs 6, 7, and  9) like that observed in humans [67, 91, 117]. This finding suggests that the point mutations likely do not cause partial loss-of-function. The stereotypical photoreceptor formation observed in the retinas of wild-type adult flies is not observed in the *Snrnp200* mutants. Instead, they possess photoreceptors that are arranged in a novel ring-like structure (Figs. 8 and 9). The *Snrnp200* mutants show progressive loss of photoreceptor function, as evidenced by abnormal ERG waveforms (Figs. 6 and 7). Except for the homozygous lethality of the *Bigfoot* allele, adult flies bearing the *Snrnp200* mutations appear healthy and show no overt phenotypic defects outside of the eye, like the human condition. Thus, Drosophila can be useful for determining the tissue-restricted phenotype of RP caused by mutations in globally expressed pre-mRNA splicing factors.

ERGs of the *Snrnp200^Bf/+^* heterozygotes showed a loss of PDA (Fig. 7), which suggests defective function of the photoreceptors that express Rh1 (R1–6). However, western analysis showed no decrease in Rh1 levels and no overt mislocalization of Rh1(Supplementary Fig. 3). Despite this normal presentation, it is possible that there are defects associated with Rh1. For example, changes in the aggregation properties of Rh1 might not be readily detectable by light microscopy. Abnormal aggregation and mislocalization of Rh1 have been observed in other forms of RP [106, 118, 119]. Thus, it remains possibile that the loss of PDA in the *Snrnp200^Bf^* mutant could be due to altered Rh1.

To gain additional insights into the cellular basis for the retinal defects caused by mutations in *SNRNP200*, the eyes of wild-type and *Snrnp200* Drosophila mutants were examined using TEM (Fig. 9). In the *Snrnp200* mutants, the rhabdomeres are spread further apart from each other compared to those of the control. This could be a result of defects in the support cells of the ommatidia that develop during the pupal stage [120]. These secondary and tertiary pigment cells surround the photoreceptor cells along their entire length [73]. Similar abnormal phenotypes to those observed in the *Snrnp200* mutants have been observed for mutations in genes that affect cell fate, which is determined through cell–cell interactions that are governed by signaling pathways [73, 74, 83, 120, 121]. For example, mutations in *fat facets* (*faf*) produce extra photoreceptor cells due to abnormal signaling that alters cell fate specification [74]. Additionally, flies with *faf* mutations have abnormal ommatidia organization and patterning that is like that observed in the flies expressing eye-specific *Snrnp200* RNAi (Fig. 3). The Drosophila gene *crumbs* (*crb)* is required to maintain cell–cell contact in the eye [122]. Mutations in *crb* cause abnormal photoreceptor spacing and cellular deterioration in which the rhabdomeres fuse together in a semi-circle [71]. Interestingly, mutations in the human orthologue of *crumbs* named *crumbs cell polarity complex component 1* (*CRB1*) cause RP [123]. These phenotypes demonstrate that disruptions in cell fate determination can lead to abnormal patterning of the ommatidia, which might be caused by altered splicing due to the mutations in *Snrnp200*. This hypothesis is supported by the down-regulation of *crumbs* in the *Snrnp200^Bf/+^* mutant compared to the control (Supplementary Fig. 5) and requires further investigation.

In addition to photoreceptor patterning defects, *Snrnp200* mutants have progressive retinal degeneration as observed in the TEM images (Fig. 9). This degeneration is remarkable, as the rhabdomeres are smaller and less defined. In addition, the stalks connecting the rhabdomeres to the photoreceptor cell body appear thinner compared to that of the control (Fig. 9). The mitochondria in the mutants have abnormal cristae and altered size (Fig. 9). Mitochondrial dysfunction is well studied in Drosophila [112, 124, 125]. Mutations in the genes *sicily, Aats-met*, and *Marf* increase oxidative stress and induce lipid droplet formation in ommatidia [112]. The accumulation of lipids cause the ommatidia to spread apart from each other due to the increase in lipids between the cells and contribute to progressive photoreceptor degeneration [112]. Consistent with mitochondrial genes playing a role, we found that *Marf* is down-regulated in the *Snrnp200^Bf/+^* mutant compared to the control (Supplementary Fig. 5), and respiration is altered (Fig. 9D). Defective mitochondria are consistent with the suppression by NAC. Of note, mitochondrial defects have been linked to human retinal disease, suggesting a generalizable phenomenon [126–130]. However, it remains to be determined if mitochondrial dysfunction is a cause or consequence of the photoreceptor dysfunction.

It is unclear how mutations in ubiquitously expressed splicing factors cause a specific retinal phenotype. One hypothesis posits that mutations in genes encoding splicing factors cause mis-splicing of retinal specific mRNA transcripts; however, strong evidence for this has not yet been established. In a zebrafish study, expression of mutant *prpf31*, encoding a component of the spliceosome, showed evidence of mis-splicing of retinal transcripts such as *transducin subunit 1* (*gnat1*), *cone-rod homeobox* (*crx*), *retinal homeobox gene 1* (*rx1*), and *retinal homeobox gene 3* (*rx3*) [131]. Interestingly, a cone specific transducin gene, *gnat2*, was not mis-spliced, possibly providing an explanation for why rod photoreceptors are affected first, then cones. In addition, this study showed a reduction in the levels of many retinal transcripts. In another zebrafish model, the orthologue of *Snrnp200* was knocked down and retinal transcripts were also found to be reduced [90]. Collectively, these data suggest that mutations in genes encoding splicing factors alter retinal specific transcripts, which might explain the tissue-specific phenotype of RP.

Another non-mutually exclusive hypothesis for the retinal-specific defects posits that mutations in genes encoding splicing factors generally cause mis-splicing of mRNAs, which when translated lead to protein truncations and improper folded proteins. An over-abundance of mis-folded proteins can cause metabolic stress (Fig. 10). The retina might be extremely sensitive to metabolic stress, as it is one of the most metabolically active tissues in the body [11, 20, 132]. Photoreceptors are constantly regenerating their outer segments and rapidly responding to changes in stimuli [133–135]. These processes depend on functional proteins and abundant ATP. If a cell is expending excess energy to breakdown aberrant mRNAs and erroneous proteins, this could lead to an overall energy depletion and cellular dysfunction. Therefore, defects in pre-mRNA splicing could exacerbate the metabolic needs in a high-demand metabolic environment, providing an explanation for retinal-specific cell death.

**Figure 10:**
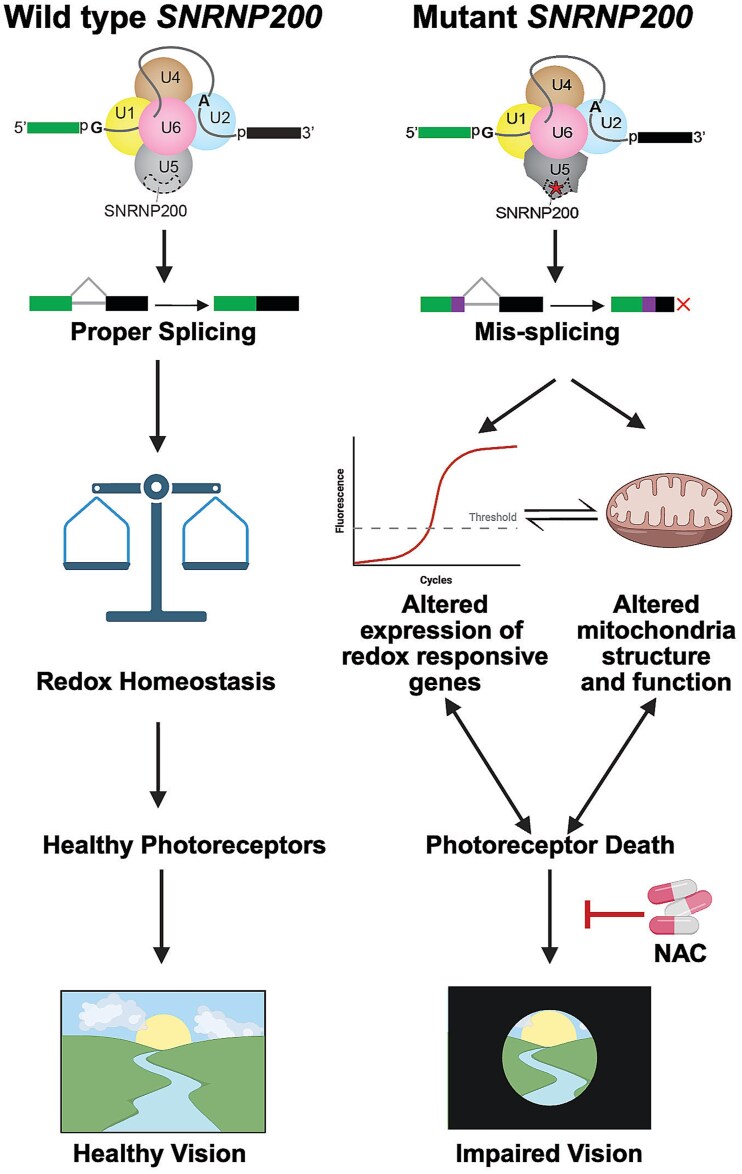
Model of mechanism of visual defects caused by *SNRNP200* mutations. Mutations in *SNRNP200* cause mis-splicing that leads to proteotoxicity and cellular stress. This causes altered mitochondrial function and activation of redox regulated genes. Collectively, these events contribute to photoreceptor apoptosis, which causes vision loss. This photoreceptor cell death can be partially suppressed by NAC treatment.

Yet, another non-mutually exclusive hypothesis for retinal specific disease due to mutations in splicing or genes could be redox imbalance (Fig. 10). In flies expressing mutant transgenes of *Prp8*, there was upregulation of stress-induced transcription factors and genes involved in glutathione metabolism [61]. Many of these are regulated by the Nuclear factor erythroid 2-related factor 2/Kelch-like ECH-associated protein 1 (Nrf2/Keap1) signaling pathway, which is activated by redox imbalance [136–138]. This finding is consistent with our data showing that NAC partially suppressed the retinal defects (Figs. 3, 8, 9). However, it remains to be determined if redox imbalance is causal for the retinal degeneration.

There is evidence that oxidative stress is at least a contributing factor in RP in humans [77, 139, 140]. Individuals with RP exhibit molecular markers of oxidative stress such as increased ROS and over-expression of antioxidant genes [23, 24, 75, 141]. NAC was used in a Phase I clinical trial for individuals with RP [142]. The results of the Phase I clinical trial showed that NAC is safe and well tolerated in patients with moderately advanced RP and may improve sub optimally functioning cones. The study reported significant improvement in best corrected visual acuity in all dosage cohorts; however, no statistical difference in central retinal sensitivity by microperimetry except for at the highest dose, and no change in mean ellipsoid zone layer width [142]. This success resulted in the Phase III clinical trial of NAC treatment for RP under the name NAC Attack which is ongoing (clinicaltrials.gov). Our study suggests that individuals with SNRNP200-RP might benefit from this clinical trial.

## Materials and methods

See Supplementary File.

## Supplementary Material

Supplementary_materials_ddag077_(2)_2
