# Supplementary material for: N-acetylcysteine suppresses retinal defects in Drosophila models of SNRNP200-associated retinitis pigmentosa
Source: Hum Mol Genet. 2026 Aug 13;35(17):ddag077. doi: 10.1093/hmg/ddag077 (PMC13648025; doi:10.1093/hmg/ddag077)
Supplement: Supplementary file 1 [file supplementary_materials_ddag077_(2)_2.zip › Supplementary_materials_ddag077 (2)/SMaterialsMethods.docx]

Supplementary - Materials and Methods

2.1 Human clinical diagnostic testing

Studies of the human patient was carried out in accordance with The Code of Ethics of the World Medical Association (Declaration of Helsinki). Human full field electroretinograms (ERGs) were conducted in a manner consistent with the International Society for Clinical Electrophysiology of Vision guidelines (65) using the Espion E2 V5 (Diagnosys LLC, Lowell, MA). Fundus photography was obtained using the Zeiss Clarus 500 (Carl Zeiss Meditec AG, Jena, Germany) fundus camera. Confirmation of the mutation in the *SNRNP200* gene was established through Sanger Sequencing of DNA isolated from blood at the University of Iowa Shivanand R. Patil Cytogenetics and Molecular Laboratory.

2.2 Drosophila stocks

Drosophila stocks were cultured in standard medium containing cornmeal and sucrose (66) at 25°C unless otherwise stated. The original *Brr2* alleles, *Brr2*^1^ and *Brr2*^e03171^ (67) used for complementation analysis were purchased from the Bloomington Stock Center (Bloomington, Indiana; 18127 and 91614, respectively). Point mutations in the *Snrnp200* gene were generated using CRISPR-mediated gene editing (WellGenetics, New Taipei City, Taiwan). Depletion of *Snrnp200* was performed using UAS-*Snrnp200* RNAi stocks [*y*^1^,*sc**,*v*^1^, s*ev*^21^; P{*y*^+t7.7^ *v*^+t1.8^;TRiP.HMS00994}attP2, #31576, #34024; and #34024; Bloomington Drosophila Stock Center (BDSC), Bloomington, IN]. Control flies expressed an RNAi transgene against *Ilp3*, a gene unrelated to eye function [*y*^1^,*sc**,*v*^1^, sev^21^; P{y^+t7.7^ *v*^+t1.8^;TRiP.HMS00546}attP2, #33681, BDSC]. To achieve knockdown of *Snrnp200*, flies from these RNAi stocks were crossed with the *GMR*-GAL4 driver stock that expresses the yeast Gal4 transcription factor in differentiated photoreceptors [*w**; P{*w*^+mc^;GAL4-ninaE.GMR}12, #1104, BDSC]. The three *Snrnp200* RNAi transgenes target different regions of the *Snrnp200* mRNA and all produced a rough eye, suggesting that the abnormal phenotype is not caused by off-target effects. Stock #34024 gave the strongest phenotype; hence it was used in subsequent analyses. When expressed by the GMR-Gal4 driver, qRT-PCR analysis of total RNA isolated from one-day-old adult heads showed a modest reduction in *Snrnp200* mRNA levels (Supplementary Fig. 6). However, caveats to this analysis include the fact that there is cell death when *Snrnp200* is knocked-down so that RNAs from those cells will not be represented in the total. In addition, the GMR-Gal4 driver is not robustly expressed in adults. For aging experiments, adults were cultured at room temperature in room lighting until the indicated timepoints (either two or seven days of adulthood). To count the rough eyed flies after drug treatment, adults were visualized by anesthetizing with CO_2_ and examining their eyes at 5x magnification using a Nikon SMZ-2B stereo microscope (Nikon Japan, Tokyo, Japan).

2.3 Quantitative Real-Time PCR (qRT-PCR)

For qRT-PCR, heads were removed from flies and immediately flash-frozen in liquid nitrogen. A minimum of three samples of ~25 heads were used per genotype. Total RNA was extracted from the heads using 350 microliters of RLT buffer with 2.86% beta-mercaptoethanol (RNeasy Mini Kit, Qiagen Sciences, Germantown, MD) and grinding with a plastic pestle in a 1.5 ml microcentrifuge tube. Following column purification per manufacturer’s recommendations, the RNA was treated with RNase Free DNase I (Qiagen Sciences, Germantown, MD). RNA purity and concentration were assessed using a NanoDrop 200 (ThermoFisher, Waltham, MA).

cDNA libraries were generated from 600 ng of total RNA/sample using the Applied Biosystems High-Capacity cDNA Reverse Transcription kit (Applied Biosystems, Waltham, MA) as 50 µL reactions. Following cDNA synthesis, cDNA was diluted with an additional 150 µL of DEPC-treated water to bring the total volume to 200 µL. Real-Time PCR was performed using Applied Biosystems Power SYBR Green RT-PCR Master Mix (Applied Biosystems, Waltham, MA) and an Applied Biosystems QuantStudio 7 Pro (Applied Biosystems, Waltham, MA) thermocycler at the Iowa Institute of Human Genetics-Genomics Division. Reactions were carried out in Applied Biosystems MicroAmp Optical 96-well reaction plates (Applied Biosystems, Waltham, MA) using 10 µL of master mix, 8 µL of cDNA template, and 1 µL each of 10 µM stocks of the forward and reverse primers. Primers (Supplementary Table 2) were generated by Integrated DNA Technologies (Coralville, IA). Data was analyzed using the ΔΔCt method and normalized using the average expression of two reference genes, *GAPDH* and *ribosomal protein L32*. Unpaired T-tests (GraphPad Prism v10.2.0, GraphPad Software, San Diego, California) were used to compare the expression of each gene in the experimental group (*Snrnp200* RNAi) to the control group (Control RNAi) across four biological replicates per group.

2.4 Retinal staining and imaging

Retinas from adult eyes were dissected, fixed, and stained using a slightly modified protocol adopted from published studies (68, 69). Briefly, seven-day-old adult flies reared in room lighting at room temperature were decapitated and bisected using a scalpel (Research Products International, Mount Prospect, IL) in cold phosphate-buffered saline (PBS) and fixed in 3.7% formaldehyde solution (Sigma-Aldrich, St. Louis, MO) for fifteen minutes at room temperature. The fixation solution was replaced with PBS and the retinas were dissected in cold 1X PBS on a SYLGARD 184 (Dow Chemical Company, Midland, MI) coated dissecting dish using superfine forceps (#17305, Bees Medical, Columbia, SC). The corneas were removed using a stainless-steel dissecting needle (#13561, Ted Pella, Redding, CA). The retinas were washed in 0.3% Triton X-100 (Dow Chemical Company, Midland, MI) in 1X PBS (PBST) at room temperature three times for ten minutes each. PBST was replaced with 5% BSA in 0.3% PBST and shaken at room temperature for twenty minutes. For primary antibody staining, 1:200 Invitrogen Texas Red-X Phalloidin (Thermo Fisher, Waltham, MA) and 1:100 4C5-s mouse anti-Drosophila Rhodopsin 1 [Developmental Studies Hybridoma Bank (DSHB) University of Iowa, Iowa City, IA] were added and incubated overnight at 4°C. The eyes were then washed with 0.3% PBST three times for ten minutes each. For secondary antibody staining, 1:400 Alexa-Fluor 488 donkey anti-mouse (Thermo Fisher, Waltham, MA) was added and incubated for two hours at room temperature. The eyes were washed with 0.3% PBST at room temperature three times for ten minutes each for the final time. The PBST was replaced with mounting media (VECTASHIELD, Vector Laboratories, Newark, CA) with DAPI and transferred to a bridge slide using a pipette. Images of tissues were taken using a Leica Dmi8 microscope with Thunder image processing (Leica Microsystems, Wetzlar, Germany).

2.5 N-acetylcysteine (NAC) treatment

A solution of 1M NAC (A7250, Sigma Aldrich, Milwaukee, Wisconsin, USA) was prepared immediately before use. NAC was added to liquified, but relatively cool, standard fly food containing cornmeal and sucrose (66) in 15 ml vials (#Z617865; Sigma Aldrich) and allowed to solidify at 17°C overnight. For the dose-response analysis, NAC was added to make final concentrations of 0.01 mM, 0.1 mM, 0.2 mM, 0.5 mM, 1 mM, 2 mM, 5 mM, 10 mM, and 20 mM. For subsequent tests, 10 mM NAC was used since it was found to be the most effective without causing death. Given that NAC is water soluble, vials with the addition of extra water served as controls. On the day after the vials were prepared, two virgin females from the GMR-GAL4 stock were added and two RNAi line males were added and then the vials were placed at 25°C. For NAC treatment of *w^1118^, Snrnp200^Bf/+^*, *Snrnp200^S108L/ S1087L^,* two virgin females and two males were added to the vials containing either NAC or extra water and then placed at 25°C. For all crosses, adults were allowed to mate for seven days at which time they were removed and discarded. As adult progeny emerged from their pupal cases, they were collected and aged in new vials with standard fly food for seven days.

2.6 Immunohistochemistry of eye/antennal imaginal discs

Drosophila third instar larval eye/antennal imaginal discs were dissected following a modified published protocol (70). In brief, larvae were dissected in a three-well glass dissecting dish in cold 1xPBS with superfine forceps (#17305, Bees Medical, Columbia, SC) by pulling the mouth hooks away from the body to remove the eye discs still attached to the mouth hooks. The eye discs were fixed in 10% neutral buffered formalin (VWR, Radnor, PA) for twenty minutes on a rotating shaker at room temperature. The discs were subjected to two five-minute washes in 0.3% Triton X-100 in 1XPBS (PBST). Following fixation, any excess tissues that remained after dissection such as cuticle and brain were removed. A portion of the mouth hooks were often left attached and used for subsequent manipulation with forceps. The tissue was then washed in 0.3% PBST for one hour at room temperature. The PBST was replaced with blocking solution [5% Bovine Serum Albumin (BSA), Research Products International, Mount Prospect, IL] in PBST for one hour at room temperature on an orbital shaker. The blocking solution was then removed and replaced with a primary antibody solution (antibody diluted in blocking solution) and placed on the orbital shaker overnight at 4°C. Following incubation, excess antibody was removed by performing three ten-minute washes in 0.3% PBST. The last wash solution was replaced with secondary antibody diluted in blocking solution and incubated at room temperature for two hours with shaking. The discs were then washed twice with 0.3% PBST for five minutes and twice with 1X PBS. Following, the discs were mounted on microscope slides (Fisherbrand Superfrost Plus slides, Thermo Fisher, Waltham, MA) in mounting media with DAPI (VECTASHIELD, Vector Laboratories, Newark, CA) and covered with a cover slip. Slides were sealed with clear nail polish and placed at 4°C for at least eight hours before imaging. Primary antibodies used were rabbit anti-cleaved Death caspase-1 (cDcp-1) (#9578, Cell Signaling Technology, Danvers, MA) at a 1:400 dilution and rat anti-lethal abnormal vision (elav) (7E8A10, Developmental Studies Hybridoma Bank, Iowa City, IA) at a 1:200 dilution. The secondary antibodies used were Alexa Fluor Goat anti-Rabbit 488 (#A-11034, Invitrogen, Waltham, MA) and Alexa Fluor Goat anti-Rat 546 (#A-11081, Invitrogen) both at a 1:400 dilution. Images of the discs were taken using a Leica Dmi8 microscope with Thunder image processing (Leica Microsystems, Wetzlar, Germany) using a 63X oil immersion lens. Images were taken as Z-stacks, deconvoluted, and projected into flat images using the Leica LASX software. Quantification on images was performed using ImageJ (National Institutes of Health, Bethesda, MD).

2.7 Antibodies Used in this study

2.8 Drosophila electroretinogram recordings

Electroretinograms were conducted following previously published procedures (71). Mutant and control adult flies (two and seven days old) were anesthetized using carbon dioxide at room temperature. The anesthetized flies were mounted into a circuit breadboard and secured to the board using a drop of melted polyethylene glycol wax that was applied to the back of the head. Filamented glass capillary tubes (1.22 mm OD, 0.68 mm ID; World Precision Instruments, Sarasota, FL) were used to make micropipette electrodes using a micropipette puller (model Pul-2; World Precision Instruments, Sarasota, FL) which were filled with a 0.7% w/v NaCl solution. Glass electrodes were placed into electrode holders containing chlorinated silver wire conductors. The tip of the reference electrode was broken to reduce resistance and inserted next to the proboscis of the fly. The recording electrode was placed into the eye. The flies were given at least 15 minutes to dark adapt before recording. The resulting signal was amplified 10X by a DC amplifier (IX1, Dagan Corporation, MI) and sampled at a rate of 10 kHz using a data acquisition device (PowerLab 26T; ADInstruments, Colorado Springs, CO). The signal was viewed on a computer monitor using LabChart Pro software (ADInstruments). Orange and blue LED lights (~1200 lux) were positioned about four centimeters from the eye and stimulated at a constant voltage. Two stimulation protocols were used for recording the receptor potential, one that used only orange light and another that used orange and blue to measure the Prolonged Depolarizing Afterpotential (PDA). The orange light only protocol used a one-second pulse of orange light at an interval of 30 seconds. The protocol to measure PDA used three consecutive one-second blue light stimuli at an interval of 30 seconds followed by multiple one-second pulses of orange light at 0.1 Hz.

2.9 Western analyses

Protein lysates were made from the heads of twelve adult flies of the same genotype and treatment group. These heads were placed in a 1.5 ml Eppendorf tube with 40 µl of a buffer containing NaCl, HEPES, KCl, and Sucrose with protease inhibitors (Protease Inhibitor Cocktail, Sigma-Aldrich, St. Louis, MO) and phosphatase inhibitors (Roche PhosStop, Sigma-Aldrich, St. Louis, MO) and 40µL of 2X Laemmli Buffer with DTT (Research Products International, Mount Prospect, IL). The heads were ground using plastic pestles to lyse the cells. The samples were incubated at room temperature for five minutes and not boiled, a departure from the standard protocol. Boiling the lysates resulted in Rh1 protein aggregation even when electrophoresed through SDS/PAGE gels. Following RT incubation, samples were centrifuged in a microcentrifuge at 15,000 rmp for five minutes at RT. The samples were divided into three aliquots of approximately 30 µL each and either used immediately or frozen at -80°C for later use.

Samples were loaded onto a precast Invitrogen NuPAGE 4-12% Bis-Tris gels (Thermo Fisher, Waltham, MA) and electrophoresed in a NuPAGE mini-gel apparatus using Invitrogen 20X MES/SDS running buffer diluted to 1X. The inner chamber of the apparatus also contained 500 μl of NuPAGE antioxidant buffer diluted in 200 ml 1X MES/SDS running buffer. The samples were electrophoresed at 95 volts for 1.5 hours. Cold 1X transfer buffer was used to transfer the proteins onto a nitrocellulose membrane (Amersham Protran 0.45µm NC, Cytiva, Marlborough, MA) using the electrophoresis apparatus (Bio-Rad Laboratories, Hercules, CA). The transfer took place using 75 volts for two hours at 4°C. The membrane was removed from the apparatus and allowed to air dry overnight at room temperature.

The membrane was rehydrated in 1X TBS and incubated in REVERT total protein stain according to the provided protocol (LI-COR, Lincoln, NE). The membrane was imaged in the LI-COR Odyssey Imager. Following REVERT stain and imaging, the membrane was incubated in blocking solution (5% BSA in 1X TBS) for one hour. The blocking buffer was replaced with primary antibody diluted in 1% BSA in 1X TBS-T and incubated overnight at 4°C. A mouse anti-Drosophila Rhodopsin 1 antibody from the Developmental Studies Hybridoma Bank (DSHB, 4C5-s. University of Iowa, Iowa City, IA) was used at 1:1000 dilution. The primary antibody solution was replaced with 1X TBS-T and washed for five minutes four times. The final wash was replaced with secondary antibody diluted in 1% BSA in 1X TBS-T and incubated at room temperature for one hour. The secondary solution was replaced with TBS-T and washed four times for five minutes then washed twice in 1X TBS for five minutes. Following the last wash, the membrane was imaged on the LI-COR Odyssey Imager. Image analysis was performed with LI-COR Image Studio software.

2.10 Transmission electron microscopy (TEM)

Drosophila retina ultrastructure was imaged following standard Electron Microscopy procedures using a Ted Pella BioWave (Ted Pella, Inc., Redding, CA) processing microwave with vacuum attachments. The tissue was immersed in 2% paraformaldehyde, 2.5% glutaraldehyde, in 0.1 M sodium cacodylate buffer at pH 7.2. After dissection the heads were incubated for three days in fixative at 4°C on a rotator. The heads were then fixed again following microwave procedures, followed by 3x Millipore water rinses, post-fixed with 1% aqueous osmium tetroxide, and rinsed again 3x with Millipore water. Concentrations from 25-100% of ethanol were used for the initial dehydration series, followed with propylene oxide as the final dehydrant. Samples were gradually infiltrated with three ratios of propylene oxide and Embed 812, followed by three changes of pure resin under vacuum. Samples were allowed to infiltrate in pure resin overnight on a rotator. The samples were embedded into flat silicone molds and cured in the oven at 62^o^C for three days. The polymerized samples were thin sectioned at 48-50 nm and stained with 1% uranyl acetate for ten minutes followed by lead citrate for one minute before TEM examination. EM grids were viewed in a JEOL 1400 Plus transmission electron microscope at 80kV. Images were captured using an AMT XR-16 mid-mount 16 mega-pixel digital camera.

2.11 Quantifying CO_2_ production with respirometry

CO_2_ production in adults was measured with respirometry (72, 73). Respirometers were constructed by gluing a 1 ml pipette tip and 50 µl capillary micropipette together. Soda lime was placed into each pipette tip and covered with a piece of foam. Flies were sorted under CO_2_ anesthesia and allowed to recover for at least 24 hours before beginning the experiment. Four adult flies of the same genotype were aspirated into each respirometer, and the top was sealed with non-hardening modeling clay. Pipette seal was monitored and any that leaked were excluded from analysis. Sixteen respirometers were hung on a custom-made rack in a latch-lid measurement chamber (one control respirometer was left empty). The chamber was filled with a red water-based dye and the chamber was closed/sealed with vacuum grease. The chamber was placed in an incubator and allowed to equilibrate at 25°C for one hour. Images were captured every 15 minutes with PhenoCapture 3.3. The liquid meniscus level was measured in each respirometer after three hours using Fiji 2.0. Data were normalized to the mean CO_2_ production of the controls. Normalized CO_2_ production values (n=5-10 animals/genotype) were compared between the *Snrnp200^Bf/+^*, *Snrnp200*^S1087L/S1087L^, *w^1118^* genotypes using a one-way ANOVA with Tukey’s multiple comparisons test in GraphPad Prism (v10.2.0, GraphPad Software, San Diego, California).

2.12 Statistical analysis

Quantification of the rough eye phenotype following NAC treatment was analyzed by screening adult females and visually scoring through a dissecting scope. The percent of adults with non-rough eyes was calculated by dividing the number of non-rough adult females by the total number of adult females screened. 40 - 120 adults were scored per genotype.

The extent of apoptosis in imaginal eye discs was analyzed using ImageJ and GraphPad Prism. Projection images were loaded into ImageJ and a threshold was set to remove background fluorescence. For quantification of the cDCP-1 signal intensity in eye discs expressing GMR-GAL4 and the *Snrnp200* RNAi, cells posterior to the morphogenetic furrow were analyzed. This was detected using staining of elav (marker of differentiated retinal cells) to mark a region of interest (ROI). Following, ImageJ was used to determine the total area of cDCP-1 staining (in the green channel) within the ROI. The percent area stained with cDCP-1 in 30-40 imaginal discs per genotype was average and compared with a Šídák's multiple comparisons test in Prism. For quantification of the signal intensity of CRISPR mutants, the whole eye disc was used.

Images of the Drosophila retinas stained with phalloidin were captured using the Leica Thunder (Leica Microsystems, Wetzler, Germany). The images were collected from the top of the retina where the top of the rhabdomeres were just in focus. The images were loaded into ImageJ and linear measurements were taken between adjacent ommatidia from the center of one ommatidia to the center of the neighboring one. Five measurements were taken per image and averaged (12-20 independent eyes per genotype). The average value for each image was then then averaged with those from the same genotype and analyzed in GraphPad Prism with Šídák’s multiple comparisons test.

qRT-PCR data was converted to relative fold changes in mRNA expression using the ΔΔCt method. Average fold changes in expression from 3-4 biological replicates were compared using either one way-ANOVA analysis with Dunnett’s multiple comparisons test or Student’s T-tests depending on number of genotypes compared.

Protein signals on westerns were analyzed using LI-COR Image Studio software (LI-COR, Lincoln, Nebraska) and GraphPad Prism (GraphPad, Boston, MA). The signal intensity of the background was subtracted from the signal of Rh1. Background signal was determined by subtracting signal from a blank lane with no protein added from the protein lanes. For protein normalization, the corrected signal of Rh1 was divided by the REVERT total signal (LI-COR, Lincoln, Nebraska) of the same lane. Then, the normalized protein signal for each genotype was compared using Šídák's multiple comparisons test.

The Prolong Depolarizing Afterpotential (PDA) was measured using the recording software LabChart Pro and GraphPad Prism (Graphpad, Boston, MA). Amplitudes were measured for each ERG recording captured in LabChart Pro. The amplitudes from flies of the same genotype and age were averaged together. The averages for two- and seven-day-old flies for each genotype were compared and the percent change among the averages were analyzed using a Šídák's multiple comparisons test in GraphPad Prism (GraphPad, Boston, MA).

The mitochondria in TEM images taken at 8,000X were measured using ImageJ. Mitochondria in each image were counted, and a circle was drawn around the area for the size measurements. Mitochondria cut off on the edges of the photos were not counted. The average mitochondrial size and number in each microscopic field were compared with Šídák’s multiple comparisons test in GraphPad Prism.
