# Supplementary material for: N-acetylcysteine suppresses retinal defects in Drosophila models of SNRNP200-associated retinitis pigmentosa
Source: Hum Mol Genet. 2026 Aug 13;35(17):ddag077. doi: 10.1093/hmg/ddag077 (PMC13648025; doi:10.1093/hmg/ddag077)
Supplement: Supplementary file 1 [file supplementary_materials_ddag077_(2)_2.zip › Supplementary_materials_ddag077 (2)/STable1-Comp.docx]

Supplementary Table 1: Lethal phase of *Snrnp200* (*Brr2*) *mutations**

| Parent 1 | | | | | |
| --- | --- | --- | --- | --- | --- |
| Parent 2 | genotype | *brr2^1^* | *brr2^e03171^* | *Snrnp200^Bf^* | *Snrnp200^S1087L^* |
|  | *brr2^1^* | embryonic/L1^#^ | embryonic/L1 | embryonic/L1 | viable |
|  | *brr2^e03171^* |  | embryonic/L1 | embryonic/L1 | viable |
|  | *Snrnp200^Bf^* |  |  | embryonic/L1 | pupal/adult^%^ |
|  | *Snrnp200^S1087L^* |  |  |  | viable |

*Greater than 200-400 progeny were scored per genotype.

^#^L1, first instar larval stage

^%^Approximately 8% of the progeny were adult escapers.
