# Supplementary material for: N-acetylcysteine suppresses retinal defects in Drosophila models of SNRNP200-associated retinitis pigmentosa
Source: Hum Mol Genet. 2026 Aug 13;35(17):ddag077. doi: 10.1093/hmg/ddag077 (PMC13648025; doi:10.1093/hmg/ddag077)
Supplement: Supplementary file 1 [file supplementary_materials_ddag077_(2)_2.zip › Supplementary_materials_ddag077 (2)/Stable2-Primerseq.docx]

Supplementary Table 2: Primers used for qRT-PCR.

| Gene Name | Forward (5’→3’) | Reverse (5’→3’) |
| --- | --- | --- |
| *crumbs* | GGATCTGCCCAAGGTGTTTA | AGTTCTTTCCAATGATGCCG |
| *CG10383* | GCCGTCATCCGAAGTTATTGG | GGGCGACTCGTACAGCAAG |
| *CG12224* | ATGTTTGACTATTCGGCTGACAA | ACCTGTAGTATGTCCACCCTG |
| *Got2* | TGTCACGGAAGCCTTCAAGAA | GTCCAGACTACGGCTCACC |
| *GstZ2* | CCGCGAGGTGAATCCAATG | CTGGGGACGTGTTTCCTCC |
| *Hpd* | ATTCGTGAGATTCGCTACCATC | GGCAATCCGTTTAGAAGGACATC |
| *Marf* | GAGACGACCACCTTTATCAACG | GCCACCTTCATGTGATCCCG |
| *Mrp4* | ACAAATCGGATTCCCTGGGAG | ACCCAAACGTGGAGTTTCATTTT |
| *Opa1* | TCAAGCTGCGATACATCGTCC | GGCAGTCCATCCTTCCATTCC |
| *Psat* | AGTTGCCCGAAGAGGTACTGA | CTGGAGCGATGGGACATCTC |
| *Shmt* | ATGGCCGATCAGAAACTGCTG | CACCGTAGTACCTCTTGCCG |
| *Snrnp200* | GAAAGCTGGACGGAACAAGGA | TCGCGCTTCTGACGTTTCA |
| *Tdh* | AGAGTAGGCCACCCAAAATTCT | GTACCGACTGACTGGGCTTTA |
