# Supplementary material for: N-acetylcysteine suppresses retinal defects in Drosophila models of SNRNP200-associated retinitis pigmentosa
Source: Hum Mol Genet. 2026 Aug 13;35(17):ddag077. doi: 10.1093/hmg/ddag077 (PMC13648025; doi:10.1093/hmg/ddag077)
Supplement: Supplementary file 1 [file supplementary_materials_ddag077_(2)_2.zip › Supplementary_materials_ddag077 (2)/SFigLegends.docx]

**Legends to Supplementary Figures**

**Supplementary Fig. 1**. Confirmation of a base change in the *SNRNP200* by Sanger sequencing. DNA was isolated from a blood sample from the patient under study here and a control individual. Sanger sequencing was performed and verified the presence of the single base change (c.2192C>T) in the *SNRNP200* gene in the patient, as shown by sequencing in the forward and reverse directions.

**Alt Text:** Sanger sequencing tracings showed the presence of the heterozygous c.2192>T base pair change.

**Supplementary Fig. 2.** The rough-eye phenotype caused by *Snrnp200* depletion in the developing eye occurs in both light and dark conditions. Flies expressing the *GMR*-GAL4 driver were crossed to those possessing the UAS-RNAi against *Snrnp200*. The graph shows the percentage of rough eye progeny that were raised in either light or dark conditions. The males show 77% and 78% percent rough eye in the light and dark, respectively, which is not statistically (ns) different from each other (*p* = 1; Fisher’s Exact). At both conditions, the females showed a 100% rough eye phenotype. The number of individual adults screened ranged from 111 to 147 for each category.

Alt Text: RNAi knock-down of *Snrnp200* in the Drosophila eye produces a rough appearance adult eye.

**Supplementary Fig. 3.** Mutations in *Snrnp200* do not cause a change in Rh1 levels. **A.** A example western analysis is shown. Rh1 was detected in protein extracts from adult heads using an anti-Rh1 antibody (green) that recognized an anticipated single band at approximately 30kD. The REVERT total protein stain (red) was used as a loading control for normalization. **B.** The intensity of the Rh1 antibody signal was quantified for each genotype relative to total protein. A range of five to seven intendent samples of protein extract from the twelve adult heads per genotype were analyzed. Šídák’s multiple comparisons test was used to determine statistical significance (GraphPad Prism Software, GraphPad, LLC Boston, MA USA). Error bars represent standard deviation of the mean. ns, non-significant **C.** Whole mounts of adult eyes were stained with antibodies to Rh1 (green) and DAPI (blue) for the indicated genotypes. No overt difference in localization was observed for Rh1 among the genotypes.

**Alt text:** Mutations in *Snrnp200* do not reduce Rh1 levels as evidenced by western analysis.

**Supplementary Fig. 4.** The *Snrnp200^Bf^/^+^* heterozygotes show apoptosis in larval imaginal eye discs that is partially suppressed by NAC. **A.** Diagram of an eye disc showing the morphogenetic furrow. Cells posterior to the morphogenic furrow are those that have differentiated. **B.** Images from larval imaginal eye discs from the indicated genotypes are shown. Images of eye discs from untreated larva are on the left and treated larvae on the right. Images of eye discs stained with antibodies to elav (red), cDCP-1 (green) and DAPI (blue) are shown. The area of quantification included the entire eye disc. **C.** Quantification of cDCP-1 signal intensity was performed on the area posterior to the morphogenic furrow. A range of 16 to 39 eye discs were analyzed per genotype. Šídák’s multiple comparisons test was used to determine statistical significance (GraphPad Prism Software, GraphPad, LLC Boston, MA USA). Error bars represent the standard deviation. ns, non-significant; *, p < 0.05; ***, p < 0.001; ****, p < 0.0001

Alt text: *Snrnp200* mutants showed death of cells in the larval eye disc that was reduced by feeding NAC.

**Supplementary Fig. 5**. Genes associated with retinal dysfunction in Drosophila are down-regulated in the *Snrnp200^Bf/+^* mutants. qRT-PCR was performed on RNA isolated from three sets of 30 heads per genotype. The ΔΔCt method was used to generate fold changes in relative expression level of each gene following normalization to reference genes *GAPDH* and *ribosomal protein L32*. Expression fold changes in the *Snrnp200* mutants were compared to a single *w^1118^* control group using a one-way ANOVA followed by Dunnett’s multiple comparisons test. Error bars represent standard deviation. ns, non-significant; *, *p* <0.05

**Alt text:** *Snrnp200* mutants down-regulate genes involved in retinal dysfunction as evidence by RT-PCR.

**Supplementary Fig. 6.** Effects of *Snrnp200* RNAi on *Snrnp200* RNA levels in adult heads. The *GMR*-Gal4 driver stock was crossed to flies possessing a *Snrnp200* RNAi transgene or control RNAi. Total RNA was isolated from heads of one-day-old adult progeny and used for qRT-PCR analysis with primers to *Snrnp200* and reference gene *GAPDH* as a control. The ΔΔCt method was used to generate fold changes in relative expression level of each gene following normalization to reference genes *GAPDH* and *ribosomal protein L32*. Expression fold changes in were compared to the control using a one-way ANOVA followed by Dunnett’s multiple comparisons test. Four to six groups of 30 heads per genotype were used for the analysis. Error bars represent standard deviation of the mean. *, *p* < 0.05

**Alt text:** RNAi knock-down of *Snrnp200* by the *GMR*-Gal4 driver causes a slight decrease in *Snrnp200* mRNA levels in adult heads. This is likely an underestimate since reduced *Snrnp200* causes cell death.
